# Insecticide susceptibility status of *Anopheles albimanus* populations in historical malaria foci in Quintana Roo, Mexico

**DOI:** 10.1186/s12936-024-04993-0

**Published:** 2024-05-25

**Authors:** Denis Escobar, Gabriela González-Olvera, Ángel S. Gómez-Rivera, Juan Navarrete-Carballo, Pedro Mis-Ávila, Raquel Baack-Valle, Guillermo Escalante, Gerardo Reyes-Cabrera, Fabian Correa-Morales, Azael Che-Mendoza, Gonzalo Vazquez-Prokopec, Audrey Lenhart, Pablo Manrique-Saide

**Affiliations:** 1 Unidad Colaborativa de Bioensayos Entomológicos, Campus de Ciencias Biológicas y Agropecuarias, Universidad Autónoma de Yucatán, Mérida, Yucatán Mexico; 2 Instituto de Investigaciones en Microbiología, Facultad de Ciencias, Universidad Nacional Autónoma de Honduras, Tegucigalpa, Honduras; 3 Servicios Estatales de Salud de Quintana Roo, Chetumal, Quintana Roo Mexico; 4 Centro Nacional de Programas Preventivos y Control de Enfermedades, Secretaría de Salud, Ciudad de México, Mexico; 5 Department of Environmental Sciences, Emory University, Atlanta, GA USA; 6 Entomology Branch, Division of Parasitic Diseases and Malaria, U.S. Centers for Disease Control and Prevention, Atlanta, GA USA

**Keywords:** Malaria, Mexico, Anopheles vectors, Insecticide resistance

## Abstract

**Background:**

Mexico has experienced a significant reduction in malaria cases over the past two decades. Certification of localities as malaria-free areas (MFAs) has been proposed as a steppingstone before elimination is achieved throughout the country. The Mexican state of Quintana Roo is a candidate for MFA certification. Monitoring the status of insecticide susceptibility of major vectors is crucial for MFA certification. This study describes the susceptibility status of *Anopheles albimanus,* main malaria vector, from historically important malaria foci in Quintana Roo, using both phenotypic and genotypic approaches.

**Methods:**

Adult mosquito collections were carried out at three localities: Palmar (Municipality of Othon P. Blanco), Buenavista (Bacalar) and Puerto Morelos (Puerto Morelos). Outdoor human-landing catches were performed by pairs of trained staff from 18:00 to 22:00 during 3-night periods at each locality during the rainy season of 2022. Wild-caught female mosquitoes were exposed to diagnostic doses of deltamethrin, permethrin, malathion, pirimiphos-methyl or bendiocarb using CDC bottle bioassays. Mortality was registered at the diagnostic time and recovery was assessed 24 h after exposure. Molecular analyses targeting the *Voltage-Gated Sodium Channel* (*vgsc*) gene and acetylcholinesterase (*ace-1*) gene were used to screen for target site polymorphisms. An SNP analysis was carried out to identify mutations at position 995 in the *vgsc* gene and at position 280 in the *ace-1* gene.

**Results:**

A total of 2828 anophelines were collected. The main species identified were *Anopheles albimanus* (82%) and *Anopheles vestitipennis* (16%). Mortalities in the CDC bottle bioassay ranged from 99% to 100% for all the insecticides and mosquito species. Sequence analysis was performed on 35 *An. albimanus* across the three localities; of those, 25 were analysed for *vgsc* and 10 for *ace-1* mutations. All individuals showed wild type alleles.

**Conclusion:**

The results demonstrated that *An. albimanus* populations from historical malaria foci in Quintana Roo are susceptible to the main insecticides used by the Ministry of Health.

**Supplementary Information:**

The online version contains supplementary material available at 10.1186/s12936-024-04993-0.

## Background

Malaria is a vector-borne disease caused by *Plasmodium* parasites transmitted to people by *Anopheles* mosquitoes [1]. Robust surveillance systems, prompt diagnosis, timely treatment of parasite-confirmed cases and the use of insecticides have been the cornerstone interventions used to achieve control and to pursue malaria elimination worldwide [2]. In Latin America, malaria cases have decreased considerably during the last decade, with 0.6 million cases reported in 2021; with active transmission persisting in the Amazon basin and the Mesoamerican region with foci in Mexico, Guatemala, the “Moskitia” region between Honduras and Nicaragua, and Panama [1, 3].

As part of malaria elimination strategies, the World Health Organization (WHO) has proposed a combination of concepts and operational definitions to adapt interventions according to local settings. This framework classifies foci and transmission risk based on the characteristics of receptivity, vulnerability and transmission intensity [4]. As malaria cases decrease, countries are advised to use epidemiological data to guide intervention strategies at a fine spatial scale. Core vector control interventions such as insecticide-treated nets (ITNs) and indoor residual spraying (IRS) are recommended in areas with recent local malaria transmission (active and residual foci) and where transmission has been interrupted for more than three years but where the risk of reestablishment is present [2, 4].

In Mexico, autochthonous malaria transmission involves *Plasmodium vivax* (100% of cases) and occurs at foci within five states with geographic, ecological and immigration characteristics that contribute to their receptivity and vulnerability [5]. The Mexican state of Quintana Roo in the Yucatan Peninsula was a historically endemic area that has seen a dramatic reduction in cases followed by sporadic transmission limited to the municipalities of Othon P. Blanco, Bacalar, Solidaridad, and Puerto Morelos [6, 7]. In 2019, the Ministry of Health in Quintana Roo performed an epidemiological stratification and classified Bacalar and Othon P. Blanco as “cleared” foci (where cases have not been reported for at least 3 consecutive years) and Puerto Morelos as a “residual-non active” focus (with transmission not reported for at least one year), as defined by the WHO [6, 8].

Pyrethroid-based interventions together with DDT (ITNs and IRS) have been used for *Anopheles* vector control in Mexico since 1955 [9, 10]. The continuous use of insecticides can lead to the emergence of insecticide resistance, particularly if only one chemical group is employed [11]. In 2012, the WHO launched a global plan for managing insecticide resistance in malaria vectors [12]. A 2018 report described the occurrence of insecticide resistance in major malaria vectors from seventy-nine countries, twelve of them being in Latin America. Countries, including Guatemala and Honduras in Central America and others in South America, described the presence of phenotypic insecticide resistance in *Anopheles albimanus* and *Anopheles darlingi*, respectively [13].

*Knock-down resistance* (kdr) mutations in the voltage-gated sodium channel (*vgsc*) gene have been reported in Neotropical *Anopheles* [14]. Two non-synonymous mutations, L995F and L995C, have been described in *An. albimanus* [15, 16]*.* In Mexico, a previous study carried out in the Yucatan Peninsula showed the presence of *An. albimanus* populations resistant to DDT, deltamethrin and pirimiphos-methyl, together with elevated levels of detoxifying enzymes (glutathione S-transferases (GSTs), cytochrome P450s and esterases) [17]; however, limited data are available regarding target-site mutations in *Anopheles* from Mexico.

The Mexican national malaria elimination plan aims to certify MFA’s after foci characterization and effective case management and responsive vector control [5, 18]. As a part of this process, persistent transmission foci in Quintana Roo have been subjected to insecticide application with deltamethrin and bendiocarb, both with IRS, during the last 5 years to maintain low vector densities and limit the chance of local transmission [4, 6]. However, even with routine insecticide-based interventions in place, recent data on insecticide susceptibility has not been generated. This study aimed to evaluate the susceptibility status (phenotypic and genotypic) of the main *Anopheles* species in historical endemic foci of malaria in Quintana Roo, Mexico, as part of malaria foci characterization for malaria elimination.

## Methods

### Study site

**Fig. 1:**
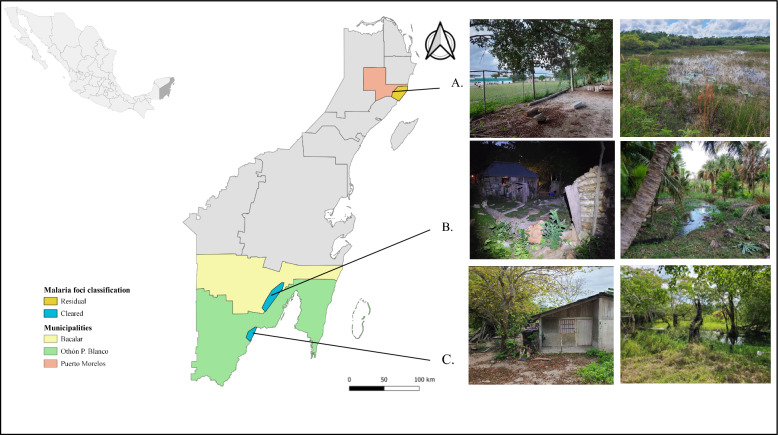
Location of the study area and localities, and general aspects of the collection sites. **A** Puerto Morelos-Puerto Morelos. **B** Buenavista-Bacalar. **C** Palmar-Othón P. Blanco, in Quintana Roo, Mexico

The municipalities of Othón P. Blanco, Bacalar and Puerto Morelos are recognized by the Ministry of Health (MoH) as historically important malaria foci in Quintana Roo. In agreement with the MoH, the principal localities from each municipality (most populated historically endemic area with anopheline mosquitoes) were selected for this study (Fig. 1): Palmar (18°26′53.8ʺN 88°31′40.8ʺW) and Buenavista (18°52′58.9ʺN 88°14′20.3ʺW) classified as “cleared” foci, and Puerto Morelos (20°51′13.2ʺN 86°53′49.4ʺW) classified as a “residual non-active” focus from the municipalities of Othon P. Blanco, Bacalar and Puerto Morelos, respectively [4, 5, 8].

Palmar and Buenavista are rural communities located close to the border with Belize. Puerto Morelos, considered an urban area (population = 26,921 inhabitants), is located 25 km from Cancun, one of the main tourist areas of Mexico. Activities related to agriculture and livestock are the main source of human activities in Palmar and Buenavista, and economic activities in Puerto Morelos are based on tourism and economic trade [19]. The temperature at the study sites oscillates between 25 and 29 °C during the rainy season (May–October) with an average of 1300 mm of rain per year [20]. The vegetation in the study areas is characterized by wetland forests, including swamps, mangroves and small ponds (Fig. 1) [21].

### Mosquito collection and identification

Outdoor human-landing catches (HLCs) were performed by pairs of trained staff from 18:00 to 22:00 during 3-night periods at each site during October 2022 (rainy season). Collections were made within the locality, 50 m. from houses and larval habitats such as ponds and small creeks (Fig. 1). All the collected *Anopheles* were placed in cages (Model BugDorm-1 Insect Rearing Cage, L31 × W31 × H9 cm; Taiwan) and maintained alive between 8 and 12 h to separate those individuals that were not in optimal physiological condition to carry out bioassays. Mosquitoes were morphologically identified using keys for the *Anopheles* of Central America and Mexico [22].

### CDC bottle bioassays

Due to the limitations of rearing an F1 generation, *Anopheles* spp. wild-caught adult mosquitoes were used for bioassays as reported previously [23]; each bioassay was carried out based on mosquito availability with at least 20 individuals tested per insecticide. Using the standard CDC bottle bioassay method, between 20 and 25 female mosquitoes per bottle in four replicates (Wheaton, USA) were exposed to the diagnostic doses of deltamethrin (12.5 µg/mL), permethrin (21.5 µg/mL), malathion (50 µg/mL), pirimiphos-methyl (50 µg/mL) or bendiocarb (12.5 µg/mL) and between 10 and 15 mosquitoes as control in a bottle coated with acetone [24]. When possible, replicates were carried out for both pyrethroids. For each insecticide, the number of dead/alive mosquitoes was registered at ten-minute intervals until 30 min of exposure was achieved (diagnostic time), and mortality was scored. Mosquitoes were then transferred to a separate holding container, provided sugar water, and held for 24 h to detect recovery. The percentage of dead mosquitoes at the diagnostic time was determined to characterize the susceptibility status of each population according to WHO criteria [25]. At the end of the bioassay, the mosquitoes were stored in tubes with 70% ethanol and transported to the Centre for Genetic Research of the Universidad Nacional Autónoma de Honduras and stored at − 20 °C for molecular analyses.

### DNA extraction and amplification

To detect target-site mutations on genes of interest, mosquitoes exposed to pyrethroids (*vgsc*) and carbamates (*ace-1*) were chosen for analysis. Given the absence of phenotypically resistant mosquitoes, a subset of five mosquitoes exposed to deltamethrin, permethrin and bendiocarb were randomly selected per site. DNA was extracted from each specimen following the Extracta protocol (Quantabio, Beverly, Massachusetts, USA). For each specimen, hind legs were dissected and placed in a 0.2 mL conical tube with 25 μL of extraction reagent. A thirty-minute lysis at 95 °C was carried out. DNA was stabilized at a final volume of 50 μL and stored at − 20 °C until further use.

Primers designed for *An. albimanus* were used to amplify segments of *vgsc* and *ace-1*. The *vgsc* target was amplified using primers AAKDRF2 (5′—AGR TGG AAY TTY CAN GAY TTY—3′) and AADKDRR2: (5ʹ—GTT CGT CTC ATT ATC C—3ʹ) [16]. PCR reactions were carried out in a volume of 50 μL, with 25 μL of Taq Master Mix 2 × (Promega, Madison, Wisconsin, USA), 2.5 μL of each primer (10 μM), 2 μL of DNA, and nuclease-free water. The PCR programme was as follows: one cycle at 95 °C for 3 min, 40 cycles at 95 °C for 45 s, 45 °C for 45 s, 72 °C for 1 min, and 1 cycle at 72 °C for 5 min in a thermocycler (Veriti, Applied Byosistems). The *ace-1* target was amplified with primers ACE1DAF: (5ʹ- TAA GAA GGT GGA CGT GTG GC -3ʹ) and ACE1DAR: (5ʹ—AGG GCA AGG TTC TGA TCG AA—3ʹ) [16]. PCR amplifications were carried out in a volume of 50 μL, with 25 μL of Taq Master Mix 2 × (Promega, Madison, Wisconsin, USA), 2.0 μL of each primer (10 μM), 2 μL of DNA, and nuclease-free water. PCR program was as follows: 1 cycle at 94 °C for 3 min, 35 cycles at 94 °C for 30 s, 61 °C for 30 s, 72 °C for 1 min, and 1 cycle at 72 °C for 10 min. PCR products were separated by electrophoresis on 1% agarose gels stained with ethidium bromide.

### Sequence analysis

Amplification products of both *vgsc* and *ace-1* were sequenced using the same primers used for PCR [16]. Purification and sequencing services were provided by Psomagen^®^ (www.psomagen.com, Maryland, USA). The sequences were edited with the Geneious^®^ 9.1.7 software (https://www.geneious.com); Auckland, New Zealand). SNP analyses were carried out at positions 995 and 280 for *vgsc* and *ace-1* sequences, respectively [24]. All sequences were submitted as queries to NCBI through the BLASTn tool under default parameters to identify the most similar sequences in the GenBank nucleotide collection.

## Results

### Mosquito species collected

A total of 2828 anophelines were collected during the study period from the three localities. Puerto Morelos yielded the largest mosquito collections (55%) (Table 1). The most abundant species collected was *An. albimanus* (87% of the total mosquitoes captured), followed by *Anopheles vestitipennis* (10%). Additionally, specimens of *Anopheles crucians* (n = 64) and *Anopheles gabaldoni* (n = 21) both in Palmar and Buenavista, and *An. darlingi* (n = 1) and *Anopheles neomaculipalpus* (n = 1) in Palmar, were collected.

**Table 1:** Total *Anopheles* collected per locality in Quintana Roo malaria foci collected by outdoor human-landing catches during October 2022 (rainy season)

Municipality	Locality	Foci status	*An. albimanus n* (%)	*An. vestitipennis n* (%)	Other *Anopheles* spp. *n* (%)	Total (%)
Othon P. Blanco	Palmar	Cleared	792 (32.3)	271 (93.7)	85 (98)	1,148 (41)
Bacalar	Buenavista	Cleared	103 (4.2)	18 (6.3)	2 (2)	121 (4)
Puerto Morelos	Puerto Morelos	Residual	1557 (63.5)	0 (0)	0 (0)	1,557 (55)
Total			2452 (86.7)	289 (10.2)	87 (3.1)	2828 (100)

### Susceptibility assays

Bioassays were carried out for all insecticides with *An. albimanus* populations from Palmar and Puerto Morelos. Due to the limited number of *An. albimanus* collected in Buenavista, it was only possible to perform bioassays with deltamethrin. In Palmar, given the availability of specimens, additional bioassays were conducted with four insecticides (deltamethrin, bendiocarb, permethrin and malathion) with *An. vestitipennis* (Table 2). *Anopheles albimanus* populations from all three localities exhibited complete susceptibility to all insecticides, with mortalities ranging from 99 to 100% for the insecticides evaluated (Table 2, Supplemental Fig. S1). Similar results were observed for *An. vestitipennis* from Palmar (Table 2).

**Table 2:** Bottle bioassay results for malaria vectors from different sites in Quintana Roo, Mexico

Species	Municipality	Locality	Insecticide	*n*	% Mortality at diagnostic time
*An. vestitipennis*	Othon P. Blanco	Palmar	Deltamethrin	80	100%
			Permethrin	35	100%
			Bendiocarb	39	100%
			Pirimiphos-methyl	58	100%
*An. albimanus*	Othon P. Blanco	Palmar	Deltamethrin	122	99%
			Permethrin	148	100%
			Bendiocarb	145	100%
			Pirimiphos-methyl	114	99%
			Malathion	111	100%
	Bacalar	Buenavista	Deltamethrin	92	100%
	Puerto Morelos	Puerto Morelos	Deltamethrin	450	100%
			Permethrin	368	100%
			Bendiocarb	108	100%
			Pirimiphos-methyl	101	100%
			Malathion	100	100%

The *An. albimanus* population from Puerto Morelos was the only population that showed recovery to deltamethrin and permethrin at 24 h post-exposure, 2% and 2.5%, respectively.

### Sequencing analysis of vgsc and ace-1 genes

Thirty-five sequences corresponding to randomly selected *An. albimanus* from the three localities were analysed, and one sequence per site was deposited in GenBank. Twenty-five sequences were analysed for mutations on *vgsc* and ten for *ace-1*. Five individuals exposed to deltamethrin and five exposed to permethrin from both Palmar and Puerto Morelos were included for *vgsc* analysis. In Buenavista, only 5 individuals exposed to deltamethrin were included for *vgsc* analysis. Five individuals each from Othon P. Blanco and Puerto Morelos exposed to bendiocarb were selected for *ace-1* analysis. The consensus sequences from samples were aligned and compared with the reference genome from *An. albimanus* (KF137581) and *An. gambiae* (AGAP004707-RA) from GenBank and Vectorbase, respectively. An SNP analysis was carried out to identify mutations at positions 995 for *vgsc* and 280 for *ace-1*. Analysis revealed that all individuals had wild type alleles.

## Discussion

According to previous reports, *An. albimanus* was the main malaria vector distributed across Quintana Roo, although other anopheline species like *An. vestitipennis* are also present and could present a risk for malaria reestablishment [25–27]. These results corroborate the predominance of *An. albimanus* as the main malaria vector species in these historical malaria foci in Quintana Roo and highlight its importance for insecticide resistance monitoring.

Malaria vectors are impacted by insecticides through vector control activities, leading to the selection of resistance in target populations [11, 14]. In addition to the use of insecticides for public health, the use of insecticides in agricultural activities constitutes an additional selection pressure [28]. The municipalities of Othon P. Blanco, Bacalar and Puerto Morelos have been areas of interest for vector control activities due to their epidemiological importance and malaria receptivity [17, 29]. These municipalities were the major areas of transmission in Quintana Roo between 2010 and 2019, where 148 out of 171 autochthonous cases at state level were reported [7, 30], and ITNs impregnated with the pyrethroid alphacypermethrin were deployed as well as IRS using bendiocarb was conducted according to local pre-elimination plans [6, 31]. Despite these interventions over the last ten years, findings here reported suggest that the local malaria vectors remain susceptible to insecticides.

A recent publication from Solis-Santoyo et al. reported *An. albimanus* populations that were resistant to deltamethrin and permethrin but susceptible to malathion and bendiocarb in two localities in Chiapas State in southern Mexico [32]. These results differ from the data presented here in two ways. First, the entomological collections in Chiapas were conducted in cattle corrals from localities with insecticide pressure derived from livestock and agriculture activities. Second, the diagnostic doses used in the bottle bioassays in the Chiapas study were established using a local reference strain, with insecticide concentrations lower than those recommended by globally recommended protocols [32–35]. National insecticide resistance data for malaria vectors is scarce, highlighting the importance of defining consistent methods for monitoring insecticide susceptibility of anophelines in Mexico.

Together with phenotypic data, this study provides evidence of the absence of *kdr* and *ace-1* mutations in *An. albimanus* from Quintana Roo*.* Bioassays are key methods for detecting phenotypic resistance in vector populations, however, they can be complemented with molecular assays to detect polymorphisms that can arise at early stages of resistance development [33, 36–38]. Target-site screening on anophelines has been carried out routinely in other regions like Africa, yet such data are relatively scarce in Latin America [13, 39, 40]. Although a small subset of samples was screened, the approach employed aligns with similar studies in Colombia and Peru, where target-site allele screening together with bioassays has been used to improve insecticide resistance surveillance and inform insecticide resistance monitoring programs [24, 41, 42].

Current vector control guidelines highlight the importance of including a plan for insecticide resistance monitoring within national malaria elimination plans to guide decision-making for vector control interventions in different transmission scenarios and achieve MFA [4, 12]. In particular, the “*Global Technical Strategy for Malaria”* states that to build a robust entomological surveillance, monitoring and evaluation programme, countries should generate data in all settings, including those that are malaria-free but at risk of reestablishment, to prevent resurgence [2]. This report highlights that insecticides, including those recommended in Mexican guidelines NOM-032-SSA2-2014, can be used for immediate response in case of a malaria outbreak and as part of a preventive residual intervention programme in areas where reestablishment of transmission is a risk [43–45].

These findings demonstrate an absence of resistance, but it’s unclear the extent to which this can be extrapolated to other localities within the State where agricultural or public health use of insecticides may be higher. A previous study in 2007 reported the presence of pyrethroid resistance on *An. albimanus* populations in three localities, including Palmar (included in this study), and where enzymatic resistance mechanisms in both Quintana Roo and Campeche (also in the Yucatan Peninsula) were detected [17]. Therefore, evaluation of *An. albimanus* populations from other potential areas/localities where insecticide pressure exists due to routine applications should be considered.

Furthermore, future studies could include evaluations with alphacypermethrin (main pyrethroid used in recent ITN campaigns) which was not evaluated here, together with other less abundant malaria vector species, such as *An. vestitipennis* and *An. darlingi* [27, 29]. Further studies should be carried out to expand the analysis to other sites and foci in the Yucatan, such as the neighbouring state of Campeche, as well as border areas with Guatemala and Belize, including both phenotypic and genotypic approaches.

To strengthen malaria elimination in Quintana Roo and the rest of the Yucatan, routine insecticide resistance monitoring should be in place together with effective vector control coverage in highest risk areas. According to guidance provided by the WHO, each malaria-endemic country should develop a national plan where sentinel sites for routine insecticide resistance monitoring are chosen; in this context, Quintana Roo could include the data provided here as baseline information and expand upon it as needs dictate, to improve integrated vector management strategies [46].

## Conclusions

Current *An. albimanus* populations in historical malaria foci from Quintana Roo remain susceptible to the main active ingredients in products used by the MoH for vector control in Mexico. Molecular analysis confirmed the absence of two main target-site mutations associated with resistance. This information could be used by local and national authorities to inform current vector control strategies and strengthen the malaria elimination and MFA certification process.

## Disclaimer

The findings and conclusions in this paper are those of the authors and do not necessarily represent the official position of the U.S. Centers for Disease Control and Prevention.

### Supplementary Information

Supplementary Material 1.

## Data Availability

All data generated or analysed during this study are included in this published article.

## Abbreviations

MFA: Malaria-free area
VGSC: *Voltage-gated sodium channel*
Kdr: Knock-down resistance
WHO: World Health Organization
CDC: Centers for disease control and prevention
SNP: Single nucleotide polymorphism
NCBI: National center for biotechnology information
