# Supplementary material for: Insecticide susceptibility status of Anopheles albimanus populations in historical malaria foci in Quintana Roo, Mexico
Source: Malar J. 2024 May 25;23:165. doi: 10.1186/s12936-024-04993-0 (PMC11128101; doi:10.1186/s12936-024-04993-0)
Supplement: Supplementary file 1 — Supplementary Material 1. [file 12936_2024_4993_MOESM1_ESM.docx]

Supplemental File




Fig. S1 Knockdown and mortality percentages with diagnostic doses at diagnostic time on wild-caught *An. albimanus* from malaria foci of Quintana Roo. OPB: Othon P. Blanco; PTM: Puerto Morelos; BAC: Bacalar.
